# Practical Suggestions Respecting the Varieties of Electric Currents and the Uses of Electricity in Medicine

**Published:** 1886-06

**Authors:** 


**Practical Suggestions Respecting the Varieties of Electric Currents and the Uses of Electricity in Medicine, with Hints Relating to the Selection and Care of Electrical Apparatus.** By AMBROSE L. RANNEY, M. D., Professor of Anatomy and Physiology of the Nervous System in the New York Post-Graduate Medical School, etc. New York: D. Appleton & Co., 1, 3 and 5 Bond street.

When we say that this book is what the title page indicates, we say all that is necessary. Such a book, containing the information essential to a scientific and successful use of electricity in medical practice, has been needed. A good part of the book has been published before, but not in book form.

